# Polycystic Ovary Syndrome, Affective Symptoms, and Neuroactive Steroids: a Focus on Allopregnanolone

**DOI:** 10.1007/s11920-021-01244-w

**Published:** 2021-04-21

**Authors:** Lindsay R. Standeven, Elizabeth Olson, Nicole Leistikow, Jennifer L. Payne, Lauren M. Osborne, Liisa Hantsoo

**Affiliations:** 1 Women’s Mood Disorders Center, Department of Psychiatry and Behavioral Sciences, Johns Hopkins University School of Medicine, Baltimore, MD USA; 2 School of Medicine, University of Maryland, Baltimore, MD USA; 3 Department of Psychiatry, University of Maryland, Baltimore, MD USA; 4 Department of Gynecology and Obstetrics, Johns Hopkins University School of Medicine, Baltimore, MD USA

**Keywords:** Polycystic ovary syndrome, Neurosteroids, Mood, Anxiety, Depression

## Abstract

**Purpose of Review:**

To provide an overview of existing studies on alterations in gonadal and neuroactive steroids (NASs) and mood symptoms among women with polycystic ovary syndrome (PCOS).

**Recent Findings:**

Recent studies have demonstrated a previously underappreciated association between PCOS and comorbid depression and anxiety. However, most studies on affective symptoms among women with PCOS have been cross-sectional, limiting our knowledge about fluctuations in symptoms over the menstrual cycle and reproductive lifespan for women with PCOS, as well as the potential interplay between NAS alterations and mood symptoms. Changes in the NAS allopregnanolone (ALLO) have been implicated in several reproductive-related psychiatric disorders (e.g., premenstrual dysphoric disorder (PMDD) and postpartum depression (PPD)) as well as in normal reproductive functioning, warranting further investigation for its potential role in the psychiatric symptoms observed in women with PCOS.

**Summary:**

Prospective studies evaluating associations between psychiatric symptoms and NAS are needed to elucidate the biological causes of the increased rates of psychiatric symptoms among women with PCOS and inform clinical treatment. ALLO, with its role in normal reproductive function, menstrual dysregulation among women with PCOS, and reproductive-related psychiatric conditions, makes it a particularly intriguing candidate for future investigation.

## Associations Between Polycystic Ovary Syndrome and Symptoms of Depression and Anxiety

Polycystic ovary syndrome (PCOS) is both common and costly. It is the most widespread endocrine disorder of reproductive-aged women globally and has a prevalence ranging from 4 to 21%, depending upon the criteria used for diagnosis and the population studied [1–6]. Costs for medical visits related to PCOS in the USA are estimated at $1.16 billion annually [7]. A syndrome of heterogenous manifestations and severity, PCOS is typically diagnosed based on exclusion of other causes and the presence of several clinical characteristics: the eponymous cystic ovaries, irregular menstrual cycles or anovulation, and hyperandrogenism with its multiple manifestations [2, 3, 8]. Sequelae include infertility and obstetrical complications; symptoms of hyperandrogenism including hirsutism, acne, and alopecia; and cardiometabolic risks including dyslipidemia, impaired glucose tolerance, insulin resistance, diabetes, and hypertension [2, 8]. Treatment has historically focused on improving reproductive function and managing symptoms such as weight gain, insulin resistance, hirsutism, and acne [3].

Less well studied is PCOS’s link to comorbid depression and anxiety symptoms. A meta-analysis of more than 3000 subjects found that twice as many women with PCOS had depressive symptoms compared to women without PCOS (36% vs. 14%), and that over four times as many women with PCOS had anxiety symptoms (42% vs. 9%) [9]. This association only strengthened when severity of symptoms was considered. The same study found that women with PCOS had moderate to severe depressive symptoms at an odds ratio of 4.18 compared to women without PCOS and moderate to severe anxiety symptoms at an odds ratio of 6.55. When restricted to clinical depressive or anxiety disorders, women with PCOS continue to be diagnosed at increased rates [10–13]. Alarmingly, one small study found that women with PCOS attempted suicide at 7 times the rate of women without PCOS [14].

Historically, increased rates of depression and anxiety symptoms in women with PCOS were thought to be sufficiently explained by struggles to have children, obesity, and signs of hyperandrogenism causing low self-esteem or other psychological stress [15–17]. These conclusions are more anchored in stereotypical social constructs of women than in a rigorous analysis using matched comparator groups [18]. When controls have been matched for factors such as BMI, family history of depression, and infertility, numerous studies have found higher rates of depression and depression symptoms in women with PCOS, a discovery that has begun to propel the search for a biochemical etiology of this association [9, 12, 15, 18, 19].

One potential nexus linking PCOS with depression and anxiety symptoms is the gonadal and neuroactive steroids (NASs), derivatives of cholesterol that have potent actions in the brain and have been implicated in all three phenomena [20]. NAS can be synthesized in the brain (where they are called neurosteroids) and in the adrenal glands or gonads, and they all derive from traditional gonadal steroids: progesterone, deoxycorticosterone, and testosterone. They are broadly classified into three groups based on their biochemical structure: (1) pregnane neurosteroids (e.g., allopregnanolone (ALLO) and allotetrahydrodeoxycorticosterone (THDOC)), (2) sulfated neurosteroids (e.g., dehydroepiandrosterone sulfate (DHEAS) and pregnenolone sulfate (PREGS)), and (3) androstane neurosteroids (e.g., androstanediol and androstanol) (see Table 1). NASs cross the blood-brain barrier and, in addition to modifying serotonergic and glutamatergic tone, modify the inhibitory γ-aminobutyric acid (GABA) system [21–23]. Different NASs can be more or less inhibitory, depending on the extent of their allosteric or direct activation and potentiation of the GABA-A receptor [24]. Psychotropic drugs can modulate NAS levels, and NAS plasma concentrations appear to change in patients with depressive syndromes [25] (for a complete review of NAS in psychiatric illness, see [25] and [26]).

**Table 1:** Selected endogenous neurosteroids by category and action at GABA-A receptor

Classification	Neurosteroids	Nomenclature	Effect at GABA-A receptor
Pregnane			
	Allopregnanolone (ALLO)	5α-pregnane-3α-ol-20-one	Potentiates
	Allotetrahydrodeoxycorticosterone (THDOC)	5α-pregnane-3α,21-diol-20-one	Potentiates
Androstane			
	Androstanediol	5α-androstan-3α,17β-diol	Potentiates
	Androstanol	3α,5α-androstenol	Potentiates
Sulfated			
	Dehydroepiandrosterone sulfate (DHEAS)	Androst-5-en-3β-ol-17-one 3β-sulfate	Inhibits
	Pregnenolone sulfate (PREGS)	5-Pregnen-3β-ol-20-one sulfate	Inhibits

Nomenclature obtained and table adopted from Reddy et al. (2010) [21]

Changes in NAS levels have been implicated in mood and anxiety symptoms at menarche, pre-menstrually, in the post-partum, and during perimenopause [24, 27–34]. NAS may also play a role in PCOS; modulation of NAS has been linked to successful treatment of some aspects of PCOS [35]. There are recent data suggesting that women with PCOS show improvement in depressive and anxiety symptoms with oral contraceptive pills (OCPs), as do some women with PMDD [36]. This supports the idea that changes in NAS across women’s reproductive cycles may underlie the observed psychiatric symptoms. However, it is not known if women with PCOS experience premenstrual exacerbations of mood symptoms, nor what percentage may have comorbid PMDD.

Most studies on affective symptoms among women with PCOS have been cross-sectional, limiting our knowledge about fluctuations in symptoms over the menstrual cycle or reproductive lifespan. To our knowledge, there has been only one prospective study following women with PCOS for the onset of depression and anxiety disorders, and it did not attempt to track menstrual patterns [13], thus limiting any conclusions about whether women with PCOS may also suffer from premenstrual exacerbation of affective symptoms. Furthermore, there are no known prospective studies following *both* hormonal fluctuations and symptoms of depression and anxiety across PCOS menstrual cycles (see Table 2). Tracing the symptoms of PCOS, depression, and anxiety across time in relation to fluctuations of gonadal steroids and NAS is necessary to explore the etiologies of their association. The remainder of this paper reviews what is known about the role of gonadal steroids and NAS in PCOS and mood and anxiety symptoms to identify targets for future research.

**Table 2:** Studies assessing symptoms of depression and/or anxiety among women with PCOS and if gonadal and NAS or timing were measured

Author(s)	Year	Outcome measures	Duration of symptom assessment	Assessed timing in menstrual cycle?	Hormones and NAS assessed	Relationship between hormones and mood symptoms assessed?
Acmaz et al. [37]	2013	QOL, BDI, BAI, SCID	Cross-sectional	No	No	N/A
Adali et al. [38]	2008	BDI and GHQ-12	Cross-sectional	Yes—between days 3 and 5	Testosterone, DHEAS	No
Amiri et al. [39]	2019	QOL	Cross-sectional	Yes—menstrual cycles interval	Testosterone, DHEAS	Yes
Annagur et al. [40]	2013	SCID	Cross-sectional	Yes—early follicular (days 2–5)	DHEAS, testosterone, 17-OH progesterone	Yes
Annagür et al. [41]	2014	BDI	Cross-sectional	No	No	N/A
Arshad et al. [42]	2012	BDI	Cross-sectional	No	No	N/A
Asik et al. [43]	2015	HADS, TEMPS	Cross-sectional	Yes—early follicular (days 2–5)	Testosterone, LH, FSH	Yes
Barnard et al. [44]	2007	Zung and PCOSQ	Cross-sectional	No	No	N/A
Barry et al. [45]	2011	HADS, aggression questionnaire and STAXI, QOL	Cross-sectional	No	Testosterone	Yes
Benson et al. [46]	2008	BDI	Cross-sectional	No	Testosterone, estradiol	No
Benson et al. [47]	2009	SCL-90-R PSDI: positive symptom distress index, BDI	Cross-sectional	No	No	N/A
Bhattacharya et al. [48]	2010	PHQ-9	Cross-sectional	No	Testosterone	Yes
Cesta et al. [49]	2017	Large Swedish registry with DSM diagnoses	Cohort retrospective analysis	No	No	N/A
Cinar et al. [50]	2011	BDI, STAI, HADS, GHQ	Cross-sectional	Yes—early follicular (days 2–5)	Testosterone	No
Davari-Tanha et al. [51]	2014	Evaluation by a psychiatrist	Cross-sectional	No	No	N/A
Deeks et al. [52]	2011	HADS, MBSRQ	Cross-sectional	No	No	N/A
Elsenbruch et al. [53]	2003	SCL-90, GSI, PSDI, PST	Cross-sectional	No	Testosterone, estradiol, cortisol	No
Ercan et al. [54]	2013	BDI	Cross-sectional	Yes—early follicular (days 2–5)	Testosterone, estradiol	Yes
Hahn et al. [55]	2005	SF-36, SCL-90-R	Cross-sectional	Yes—early follicular (days not specified)	Testosterone	Yes
Harmanci et al. [56]	2013	BSI, BSRI	Cross-sectional	No	No	N/A
Hart et al. [57]	2015	Psych dx per medical record	Cohort retrospective analysis	No	No	N/A
Himelein et al. [58]	2006	BDI-SF	Cross-sectional	No	No	N/A
Hollinrake et al. [12]	2007	PRIME-MD PHQ and BDI	Cross-sectional	No	Testosterone, DHEAS, 17-OH progesterone	No
Hung et al. [59]	2014	Psychiatric diagnoses by chart	Cohort retrospective analysis	No	No	N/A
Hussain et al. [60]	2015	SCID-MINI	Cross-sectional	No	No	N/A
Jedel et al. [61, 62]	2010,2011	(MADRS-S, BSA-S, CPRS-S-A)	Cross-sectional	No	Testosterone, DHT, DHEA, androstenedione estrone, estradiol	Yes
Kerchner et al. [13]	2009	PRIME-MD PHQ (BDI) (BAI)	Prospective longitudinal—22 months between surveys, two time points	No	No	N/A
Klimczak et al. [63]	2015	BDI, PHQ-9, QIDS-SR16	Observational	No	Testosterone, DHEAS, androstenedione, 17-OH progesterone	Yes
Laggari et al. [64]	2009	BDI, STAI, *The Stressful Life Events Schedule*	Cross-sectional	Yes	No	N/A
Mansson et al. [14]	2008	MNI NPI by a psychiatrist	Cross-sectional	No	Testosterone	No
Moran et al. [65]	2015	CES-D	Cross-sectional	No	Testosterone	No
Moran et al. [66]	2012	HADS	Cross-sectional	No	No	N/A
Moran et al. [67]	2010	HADS	Cross-sectional	No	No	N/A
Naqvi et al. [68]	2015	PHQ-9	Cross-sectional	No	Testosterone	No
O ¨zenli et al. [69]	2008	BDI, STAI, Ways of Coping Inventory (WCI)	Cross-sectional	No	No	N/A
Rahiminejad et al. [70]	2014	BDI and interview with a psychiatrist	Cross-sectional	No	Testosterone	Yes
Rassi et al. [71]	2010	SCID-MINI	Cross-sectional	No	Testosterone	No
Rocco et al. [72]	1991	Minnesota Multiphasic Personality Inventory (MMPI), and STAI	Cross-sectional	No	No	Yes
Sayyah-Melli et al. [73]	2015	Screened with MMPI. Confirmed by psychologist–DSM IV	Cross-sectional	No	No	N/A
Shi et al. [74]	2011	SCL-90	Cross-sectional	No	No	N/A
Soyupek et al. [75]	2008	BDI	Cross-sectional	No	Testosterone, estradiol, DHEAS	No
Weiner et al. [76]	2004	DACL, STAI,	Cross-sectional	Yes—day 7 of cycle	Testosterone, estradiol, progesterone	Yes

## Gonadal and Neuroactive Steroids Implicated in Depression, Anxiety, and PCOS

Many studies demonstrate alterations in gonadal steroids (namely testosterone and progesterone) and NAS in women with PCOS [77–80]. Given that hyperandrogenism is a pathognomonic feature of PCOS, the majority of studies have focused on the role of altered androgens and their products (e.g., testosterone, DHEA and DHEA-S, androstenedione, and androstenediol). Average levels of testosterone and androstenedione can be higher in women with PCOS as compared to healthy controls [80, 81]. Similarly, many PCOS patients have higher DHEA-S levels [40]. These combined pathological biochemical findings have elicited curiosity about whether alterations in select gonadal steroids and NAS may underlie the greater risk for depression and anxiety symptoms observed in women with PCOS. We will therefore provide an overview of the existing studies on alterations in gonadal and NAS and mood symptoms among women with PCOS (below and in Table 1).

### Testosterone

Since alterations in testosterone levels are common in PCOS, most studies have focused on the association between aberrations in testosterone levels and mood symptoms. To date, there is conflicting evidence on the association between depressive symptoms and testosterone levels. In Jedel et al.’s (2011) study of 72 women with PCOS, circulating concentrations of testosterone were lower in women with PCOS with symptoms of depression as compared to women with PCOS without symptoms of depression [63]. Yet, another study found a positive correlation between elevated testosterone and depressive symptoms [82]. A study by Weiner and colleagues demonstrated a curvilinear relationship between testosterone levels and depression in women with and without PCOS; i.e., the most severe depression was associated with levels below and above the normal range [76]. A 2013 study by Annagür et al. did not observe differences in testosterone levels between depressed PCOS and non-depressed PCOS women [40]. Other studies found no association between testosterone and depression in women with PCOS [39, 45, 83], and some even show an inverse correlation between free testosterone and depressive symptoms [61, 84]. The evidence on testosterone levels and depressive symptoms in women with PCOS remains inconclusive, necessitating further research on alternative NASs that may play a role.

### DHEA-S

Evidence implicating DHEA-S (the sulfated form of DHEA and an excitatory NAS) in the increased risk of depression in PCOS women is also mixed. Comparing PCOS patients with major depressive disorder (MDD) to PCOS patients without MDD or other psychiatric illness, Annagür and colleagues found that DHEA-S levels were significantly higher in the depressed PCOS group [40]. On the other hand, in a review by Dokras et al. and study by Hollinrake et al., there were no significant differences in adrenal androgen DHEA-S levels between depressed women with PCOS and non-depressed women with PCOS [12, 16]. Amiri and colleagues found no significant relationship between DHEA-S levels and psychosocial-emotional status or quality of life, except for depressed sexual function [39]. No other human studies to date have directly compared DHEA-S levels in depressed versus healthy PCOS women.

### Androstenediol

Levels of androstenediol, a reduced metabolite of testosterone and androstenedione, are known to be elevated in women with PCOS [79]. Similarly, elevated levels of androstenedione have been observed [78, 79]. Jedel et al. (2011) reported diminished levels of androstenediol among women with PCOS and depression [61]. No other papers have been published on the relationship between androstenediol levels in PCOS women and mood symptoms.

### Progesterone and 17-OH Progesterone

Comparing PCOS patients with MDD to PCOS patients without any diagnosed disorder, 17-OH progesterone, a metabolite of progesterone, was significantly higher in the depressed PCOS group [61]. A more recent study by Klimczak and colleagues similarly reported that depressed patients with PCOS had greater progesterone levels than non-depressed PCOS patients [63]. No other human studies have directly compared these levels in depressed versus healthy PCOS women. Two studies have demonstrated generally elevated levels of 17-OH progesterone in PCOS women, but analysis was not specific to women diagnosed with mood disorders [79, 80].

### Allopregnanolone

ALLO, the 3α,5α-reduced metabolite of progesterone, is produced and released from the adrenal glands and ovaries following adrenocorticotropic hormone (ACTH) and gonadotropin-releasing hormone (GnRH) stimulation [85]. ALLO levels fluctuate over the reproductive lifespan: with plasma levels changing across the menstrual cycle (highest during the early luteal phase), across pregnancy (highest in the third trimester), and during the menopausal transition (fluctuating, but declining with time). ALLO is also a potent allosteric modulator of the GABA-A receptor that has significant anxiolytic effects [24, 86, 87]. Given its dual role in both reproductive functioning and the neuromodulation of stress and anxiety, it is not surprising that alterations in ALLO have been implicated in psychiatric illness at times of reproductive transition, namely postpartum depression (PPD) [41–51], PMDD [37–40, 88–94], and perimenopausal depression [52–58].

Evidence over the past decade suggests that ALLO plays a critical role in normal menstrual cycling [35, 79, 81, 95], and alterations in ALLO may contribute to the reproductive pathology observed in women with PCOS [88, 96–98]. Given that women with PCOS do not demonstrate typical menstrual cycling, it is possible that alterations in ALLO levels may be related to their ovarian dysfunction. Animal models support the notion that *elevated* ALLO may underlie the pathology observed in PCOS. Following intracerebrovascular injection of ALLO into rats, Guiliani et al. (2011) found an increase in LH release [89]; changes in ovarian steroid production and ovarian morphology; decreased follicular development; and reduced ovulation—the same phenotypic profile observed in women with PCOS [90, 91].

Evidence from clinical research studying ALLO levels among women with PCOS has been mixed, with some studies supporting elevated levels of ALLO among women with PCOS [35, 78], another showing low ALLO levels [88], and one showing no differences in ALLO levels between PCOS women and controls [81]. Treatment trials of metformin (an oral anti-hyperglycemic found to reduce androgen levels, improve insulin resistance, and restore menstrual frequency and fertility in many women with PCOS) also support alterations in ALLO levels as well as a dysregulation of the normal signaling pattern among GnRH, LH, ACTH, and ALLO, though not always in the same direction. Genazzani et al. (2010) found lower ALLO levels in PCOS women compared to healthy controls prior to treatment with metformin, and speculated that these lower levels may be responsible for observed elevations in psychiatric symptoms [88, 92]. Ganzzanie et al. (2006), on the other hand, found that following 6 months of treatment with metformin participants demonstrated significant *decreases* in androgen, LH, and ALLO levels and restored LH and ALLO coupled release [35]. In one of the only known intervention studies for mood, Hahn et al. (2006) found a significant improvement in quality of life, depression, anxiety, and aggression measures following 6 months of metformin treatment [93], though changes in NAS (including ALLO) were not assessed.

In addition to alterations in absolute ALLO levels, altered sensitivity at ALLO receptor targets (GABA-A) may contribute to the etiology of PCOS [78]. Among 9 women with PCOS, ALLO levels were elevated compared to overweight non-PCOS and normal weight non-PCOS controls. Interestingly, the PCOS women also had lower sedation scores (measured by saccadic eye velocity) following intravenous injection of ALLO, indicating reduced GABA-A receptor sensitivity. This finding suggests that the observed elevations in total ALLO among women with PCOS may be a compensatory response to reduced receptor sensitivity rather than the cause of dysregulation. No studies to our knowledge, however, have measured a relationship among alterations in GABA-A receptor sensitivity, ALLO levels, and psychiatric symptoms among women with PCOS.

## Discussion

For too long, PCOS has been viewed as an endocrine disorder without adequate attention to the interface between observed hormonal alterations and psychiatric symptoms. Though research over the past decade has been critical in confirming the increased psychiatric burden experienced by women with PCOS, additional studies are needed to characterize adequately the course of psychiatric symptoms and their relationship to biology. Indeed, many questions remain. Do women with PCOS experience premenstrual exacerbation of psychiatric symptoms? Or, conversely, do they have persistently elevated symptoms due to a lack of menstrual cycling and hormonal fluctuations? Does the severity or type of psychiatric symptoms differ across the different PCOS phenotypes (e.g., those with hyperandrogenism versus not)?

At present, studies investigating the biological causes of the psychiatric symptoms among women with PCOS are limited. Most studies have measured NAS cross-sectionally, in order to diagnose and classify PCOS subtypes, rather than for the purpose of assessing associations with psychiatric symptoms. Of the studies that have assessed NAS and mood symptoms, most have also done so only cross-sectionally (limiting longer term conclusions), focused solely on depressive symptoms (limiting our appreciation of other symptoms and diagnoses), and focused on androgens, an approach that does not apply across all PCOS phenotypes. Larger prospective studies measuring associations among psychiatric diagnoses, psychiatric symptoms, and all three classes of NAS are needed to guide future research.

To our knowledge, there are no studies prospectively and concurrently measuring both ALLO levels and psychiatric symptoms among women with PCOS. Given recent research revealing ALLO’s role in 3 important phenomena—normal reproductive functioning, menstrual dysregulation among women with PCOS, and psychiatric symptoms among women with PMDD, PPD, and perimenopausal depression—ALLO is an ideal candidate linking both the psychiatric symptoms and menstrual dysregulation observed in women with PCOS. Will we find that total ALLO levels or ALLO target dysregulation (e.g., changes in receptor plasticity, gene expression, or receptor sensitivity) underlie symptoms of depression or anxiety in women with PCOS? Are the symptoms caused by a paradoxical sensitivity to ALLO (as previously suggested among women with PMDD), reduced GABA-A receptor sensitivity (perhaps due to persistently elevated ALLO), an inability to upregulate or modulate GABA-A receptor subunits (as may be the case in PPD), or reduced total ALLO levels (due to lack of ovarian production during anovulatory cycles)? Will alterations in ALLO be entirely unrelated to the psychiatric symptoms manifested?

Improved understanding of the biological etiology of psychiatric symptoms for PCOS women is also critical in helping to determine adequate treatment approaches. For example, while OCPs remain the treatment of choice to regulate the menstrual cycle for women with PCOS, few studies have assessed their effect on psychiatric symptomatology in this population. Similarly, while metformin is used to enhance insulin sensitivity, it remains unknown if metformin, through modification of NAS levels, may improve psychiatric symptoms. If so, should it be prescribed as part of standard psychiatric practice for women with PCOS? Lastly, there are no studies to date assessing differences in psychotropic responsivity among women with PCOS. Are lower SSRI doses needed for symptom remission (as is the case for many women with PMDD) [94], or do women with PCOS respond similarly to patients with MDD or generalized anxiety disorder?

Answering these questions is critical to raising awareness of the psychiatric needs of this vulnerable population and advocating for improved screening and treatment of psychiatric symptoms by colleagues in internal medicine, family medicine, endocrinology, and gynecology, who often provide frontline care to women with PCOS. Without increased screening and education about high rates of psychiatric comorbidity, many women with PCOS may be left untreated. A first step to providing comprehensive care will be longitudinal research studies examining the links among PCOS, depression, anxiety, and neuroactive steroids. Only once we understand this intricate relationship will PCOS be viewed and treated as the complex endocrinological and psychiatric syndrome it is.
